# A Cohort Study: Comorbidity and Stage Affected the Prognosis of Melanoma Patients in Taiwan

**DOI:** 10.3389/fonc.2022.846760

**Published:** 2022-03-03

**Authors:** Chin-Kuo Chang, Yih-Shou Hsieh, Pei-Ni Chen, Shu-Chen Chu, Jing-Yang Huang, Yu-Hsun Wang, James Cheng-Chung Wei

**Affiliations:** ^1^ Institute of Medicine, Chung Shan Medical University, Taichung, Taiwan; ^2^ Department of Biochemistry, School of Medicine, Chung Shan Medical University, Taichung, Taiwan; ^3^ Institute and Department of Food Science, Central Taiwan University of Science and Technology, Taichung, Taiwan; ^4^ Center for Health Data Science, Chung Shan Medical University Hospital, Taichung, Taiwan; ^5^ Department of Medical Research, Chung Shan Medical University Hospital, Taichung, Taiwan; ^6^ Division of Allergy, Immunology and Rheumatology, Chung Shan Medical University Hospital, Taichung, Taiwan; ^7^ Graduate Institute of Integrated Medicine, China Medical University, Taichung, Taiwan

**Keywords:** melanoma, comorbidity, stage, mortality rate, survival, prognosis

## Abstract

**Background:**

Comorbidities and stages may influence the prognosis of melanoma patients in Taiwan and need to be determined.

**Methods:**

We performed a retrospective cohort study by using the national health insurance research database in Taiwan. Patients with a primary diagnosis of melanoma by the Taiwan Cancer Registry from 2009 to 2017 were recruited as the study population. The comparison group was never diagnosed with melanoma from 2000 to 2018. The Charlson comorbidity index was conducted to calculate the subjects’ disease severity. The Cox proportional hazards model analysis was used to estimate the hazard ratio of death.

**Results:**

We selected 476 patients, 55.5% of whom had comorbidity. A higher prevalence of comorbidity was associated with a more advanced cancer stage. The mortality rate increased with an increasing level of comorbidity in both cohorts and was higher among melanoma patients. The interaction between melanoma and comorbidity resulted in an increased mortality rate.

**Conclusion:**

An association between poorer survival and comorbidity was verified in this study. We found that the level of comorbidity was strongly associated with mortality. A higher risk of mortality was found in patients who had localized tumors, regional metastases, or distant metastases with more comorbidity scores. Advanced stage of melanoma patients with more comorbidities was significantly associated with the higher risk of mortality rate.

## Introduction

Cutaneous melanoma is now regarded as the fifth most common cancer in the United States. It was estimated that there were approximately 96,480 new cases, and 7,230 deaths due to melanoma of the skin will be newly diagnosed in 2019 (1). Despite being the deadliest form of skin cancer with a high mortality rate, many melanoma patients are cured after surgical excision of their primary tumor at an early stage; however, some still relapse after surgical excision. It is locally invasive and frequently spread out to regional lymph nodes and remote organs, including lung, liver, bone, and brain (2). The most common risk factors for malignant melanoma include family history, multiple moles, ultraviolet radiation, fair skin, and immunosuppression (3, 4). The incidence rate of melanoma has been increasing by between 3% and 7% per year globally for Caucasians (5). The incidence rate (less than 1/100,000) was lower in residents of Asia, including China, India, Singapore, and Japan (6). In Taiwan, the age-adjusted rate in 2006 for aggressive melanoma was 0.65/100,000 (0.71/100,000 for men, and 0.58/100,000 for women). The age distribution plot showed a peak among melanoma patients aged 70–79 years. According to the investigation of the incidence of melanoma from 1997 to 2008 by year and sex, the yearly incidence ranged from 0.66 to 1.24 (mean: 0.9) cases per 100,000 people in Taiwan (7). Excessive exposure to ultraviolet radiation is not the risk factor for most Taiwanese melanoma cases. In addition, 58% of cutaneous melanoma belongs to acral lentiginous melanoma. Advanced disease is found in 50% of cases (8).

Studies on various prognostic factors affecting melanoma survival have been reported frequently. Age has been proven to be a very strong and independent predictor of survival outcome after accounting for all the dominant prognostic factors (9). Sentinel node biopsy is an important prognostic factor in melanoma (10). Lower socioeconomic status (SES) was associated with a decreased median survival time in a statistically significant amount for all stages of melanoma (11). Men have a greater risk of having advanced disease with a poorer outcome (12). Studies have shown that the presence of a melanoma on an axial site conferred a worse prognosis than an extremity site (9). Older age and advanced stage have significant negative effects on the survival of mucosal melanoma (13). The mitotic rate is a continuous prognostic variable. It is a strong independent predictor of outcome and should be assessed and recorded in all primary melanomas including in both initial and excision biopsies (14). Level of invasion has prognostic significance in univariate analysis and remains an independent predictor of outcome in more contemporary analyses. Clinical parameters such as age, sex, skin color, pigmentation status of the tumor, and site of the primary tumor play an important role for the outcome of patients. Ulceration is an adverse prognostic factor of melanoma (15). Stage and anatomic site, but not thickness (i.e., Breslow depth), race, or ethnicity, determine prognosis of mucosal melanomas (16). Stage, male gender, and age are associated with overall survival, along with SES and the presence of multiple comorbidities (17–19). Taken together, various prognostic factors affecting melanoma survival interact mostly synergistically. The prognostic influence of comorbidity and stage on melanoma has also caused many scientists to make many valuable reports. The prevalence of chronic disease in patients with melanoma varies from 19% to 80% (20). The presence of chronic conditions prevents physicians from aggressive treatment for melanoma patients, thereby increasing the mortality rate (20). The prevalence of multiple chronic conditions increased with age (21). The majority of studies from a systematic review reported decreased chemotherapy use (75%) and inferior survival (69%) for patients with comorbidities compared to those without comorbidities (22). Patient comorbidity has a substantial effect on the cancer stage at diagnosis (23). In a cohort study conducted using Danish registry data of 23,476 melanoma patients, Grann et al. (20) reported a higher prevalence of comorbidity associated with more advanced cancer stages. Similar results were acquired by Gonzalez et al. (24) using data from the Florida State Tumor Registry (N = 32,074) in 1994 on colorectal, melanoma, breast, and prostate cancers. Comorbidity was associated with late-stage diagnosis in all four cancers, with the odds of 62% for late-stage melanoma (24). In Taiwan, just a few reports paid attention to malignant melanoma (25–27). Our current study aims at clarifying how comorbidity and stage may affect the prognosis of Taiwan melanoma patients from 2009 to 2017.

## Materials and Methods

### Data Source

This study used the data from the National Health Insurance Research Database (NHIRD) and Taiwan Cancer Registry (TCR) that was constructed by the Health and Welfare Data Science Center (HWDSC). The NHIRD covered more than 99.99% of Taiwan’s population of 23 million people in Taiwan. Two million beneficiaries were randomly sampled from the NHIRD in 2000. To protect patients’ privacy, according to the “Personal Information Protection Act” and “Non-disclosure agreement of NHIRD,” the original identification numbers were not disclosed. Data benefits include disease diagnosis of outpatient, inpatient, emergency medical claims, and cancer staging. The longitudinal characteristic of NHIRD allows researchers to identify a cohort based upon diagnoses and drug utilization, to trace the medical history, and to disclose clinical outcomes and related complications (28). TCR (29) is a nationwide population-based cancer registry system that provides detailed information on all cancer patients in Taiwan. Hospitals with more than 50-bed capacity that could supply outpatient and hospitalized cancer care are required to join in informing all newly diagnosed malignant neoplasms to the registry. In order to measure cancer care methods and treatment outcomes in Taiwan, the TCR constructed a long-form database with cancer staging and detailed treatment and recurrence information. Moreover, the long-form database included detailed information regarding cancer site-specific factors, such as laboratory values, tumor markers, and other clinical data related to patient care (30). The study had been approved by the ethical review board of the Chung Shan Medical University Hospital (CS1-20201) in Taiwan.

### Study Design and Outcome

**Figure 1:**
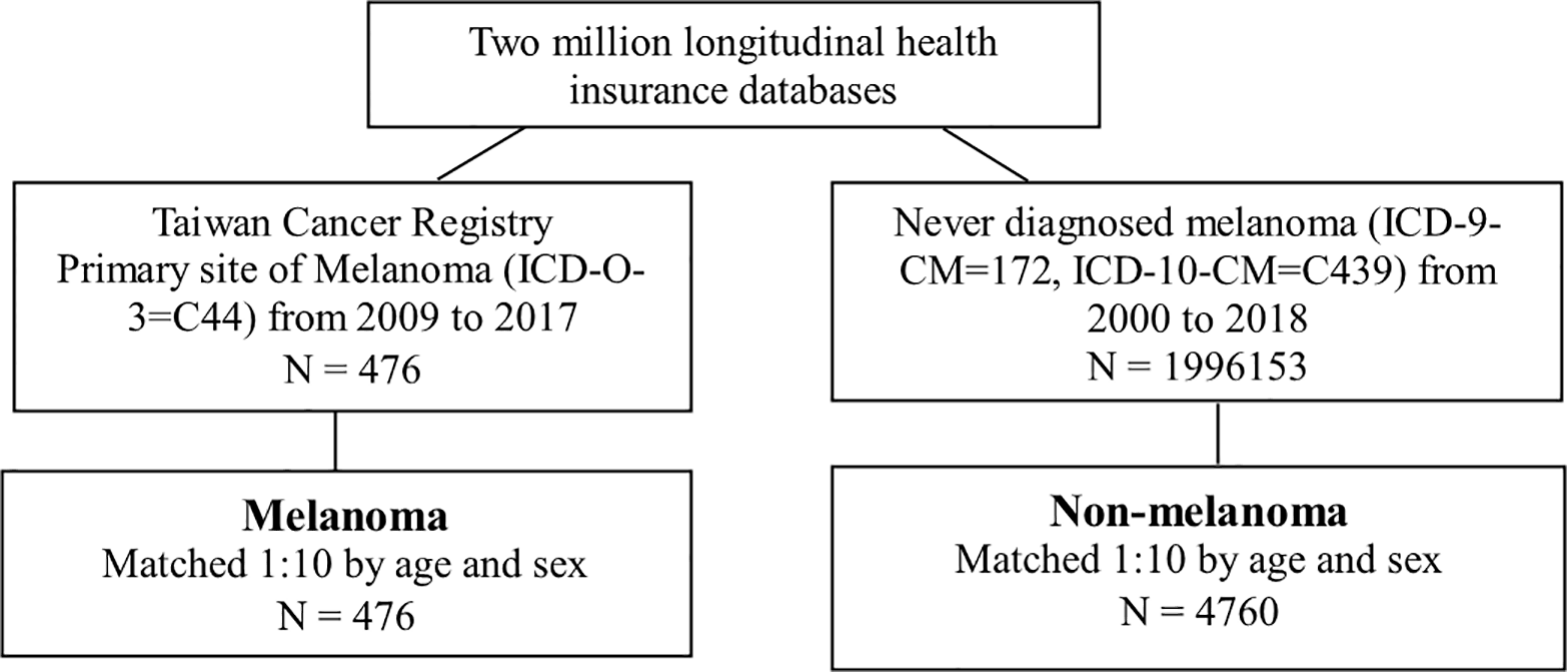
Identified Melanoma and Non-melanoma groups.

This study used a retrospective cohort study design. The study population was the primary site of melanoma (ICD-O-3=C44) from 2009 to 2017 in the TCR. The index date was admitted with the first diagnosis date of melanoma. The comparison group was defined as never diagnosis of melanoma (ICD-9-CM=172, ICD-10-CM=C439) from 2000 to 2018 (**Figure 1**). Due to the consistent index date between the melanoma group and the non-melanoma group, a 1:10 age and sex matching was conducted. The outcome variable was all-cause mortality. Both groups were followed up until the onset of death, or December 31, 2018, whichever occurred first.

The demographic variables included age, sex, monthly income, and residential region. We classified diagnoses of chronic diseases into 17 categories based on a modified version of the Charlson comorbidity index (CCI) (31). The 1 score of weight included myocardial infarction, congestive heart failure, peripheral vascular disease, cerebrovascular disease, dementia, chronic pulmonary disease, connective tissue disease, peptic ulcer disease, mild liver disease, and diabetes without chronic complication. The 2 scores of weight included diabetes with chronic complication, hemiplegia or paraplegia, and renal disease. The 3 scores of weight included any malignancy, including lymphoma and leukemia, except malignant neoplasm of skin, and severe liver disease. The 6 scores of weight included metastatic solid tumor and AIDS/HIV (**Supplementary Table 1**). The higher CCI-weighted total score represented the higher severity of the disease. The baseline characteristics also included age, sex, hypertension (ICD-9-CM=401-405, ICD-10-CM=I10-I15), and hyperlipidemia (ICD-9-CM=272, ICD-10-CM=E78). Those comorbidities were defined 1 year before the index date and at least two outpatient visits or once hospitalization.

### Statistical Analyses

The comparison of continuous and categorical variables was done using Student’s t-test, chi-square test, or Fisher’s exact test as appropriate between melanoma and non-melanoma groups. Kaplan–Meier analysis was conducted to calculate the all-cause mortality among the two groups. The log-rank test was used to test the significance. The multivariate Cox proportional hazards model was used to estimate the hazard ratios (HRs) of death. We used a time-dependent variable to assess the proportional hazards assumption. The p value was 0.2805 that it did not violate the proportional hazards assumption. For the unmeasured confounding factor, we performed E-value to define the minimum strength of the association for an unmeasured confounding effect between melanoma and non-melanoma groups (32, 33). The statistical software was SAS version 9.4 (SAS Institute Inc., NC, USA).

## Results

### Study Population

We identified 476 melanoma patients and 4,760 members of the matched comparison cohort. The majority were men (56.7%), and more than half (85%) were older than 55 years. According to tumor stage, 52 (10.9%) *in situ*, 398 (83.6%) had a localized tumor, 8 (1.7%) had regional metastases, and 18 (3.8%) had distant metastases (**Table 1**).

**Table 1:** Demographic characteristics of melanoma and non-melanoma.

	Non-melanoma (N = 4,760)		Melanoma (N = 476)		p value
	n	%	n	%	
**Age**					1
≤54	710	14.9	71	14.9	
55–69	1,430	30.0	143	30.0	
≥70	2,620	55.1	262	55.1	
Mean ± SD	69.91 ± 15.66		69.91 ± 15.67		1
**Sex**					1
Female	2,060	43.3	206	43.3	
Male	2,700	56.7	270	56.7	
**Monthly income (NT$)**					0.5469
<20,000	1,527	32.1	144	30.3	
20,001–40,000	2,349	49.3	235	49.4	
>40,000	884	18.6	97	20.4	
**Residential region**					0.0014
Northern	1,989	41.8	242	50.8	
Central	1,116	23.4	87	18.3	
Southern	1,212	25.5	111	23.3	
Others	443	9.3	36	7.6	
**Stage of melanoma**					
0			52	10.9	
I			292	61.3	
II			106	22.3	
III			8	1.7	
IV			18	3.8	
**Hypertension**	1,997	42.0	242	50.8	0.0002
**Hyperlipidemia**	986	20.7	108	22.7	0.3123
**Categories of Charlson comorbidity index score**					
Myocardial infarction	44	0.9	5	1.1	0.7854
Congestive heart failure	197	4.1	14	2.9	0.205
Peripheral vascular disease	56	1.2	8	1.7	0.3398
Cerebrovascular	444	9.3	56	11.8	0.0845
Dementia	175	3.7	18	3.8	0.9077
Chronic pulmonary disease	484	10.2	63	13.2	0.0370
Connective tissue disease	26	0.5	4	0.8	0.4176
Peptic ulcer disease	473	9.9	58	12.2	0.1214
Mild liver disease	187	3.9	33	6.9	0.0018
Diabetes without chronic complication	820	17.2	83	17.4	0.9079
Diabetes with chronic complication	244	5.1	26	5.5	0.7519
Hemiplegia or paraplegia	19	0.4	3	0.6	0.4454¶
Renal disease	310	6.5	41	8.6	0.0806
Any malignancy	50	1.1	21	4.4	<0.0001
**Charlson comorbidity index score**					<0.0001
0	2,663	55.95	212	44.5	
1	1,024	21.51	126	26.5	
2	478	10.04	54	11.3	
3	280	5.88	45	9.5	
≥4	315	6.62	39	8.2	

¶Fisher’s exact test.

### Prevalence of Comorbidities

Measurement of comorbidity resulted in 212 (44.5%) melanoma patients with comorbidity score 0, 126 patients (26.5%) with comorbidity score 1, and 54 (11.3%) with comorbidity score 2. In the more severe comorbidity categories, 45 (9.5%) had a comorbidity score 3, and 39 (8.2%) had a comorbidity score ≥4. Diabetes without chronic complication was the most prevalent comorbidity, diagnosed in 83 patients (17.4%) (**Table 1**). Chronic pulmonary disease was diagnosed in 63 patients (13.2%), followed by peptic ulcer disease (58 patients, 12.2%). The majority of melanoma patients were diagnosed with the localized stage (**Table 1**).

### All-Cause Mortality

**Figure 2:**
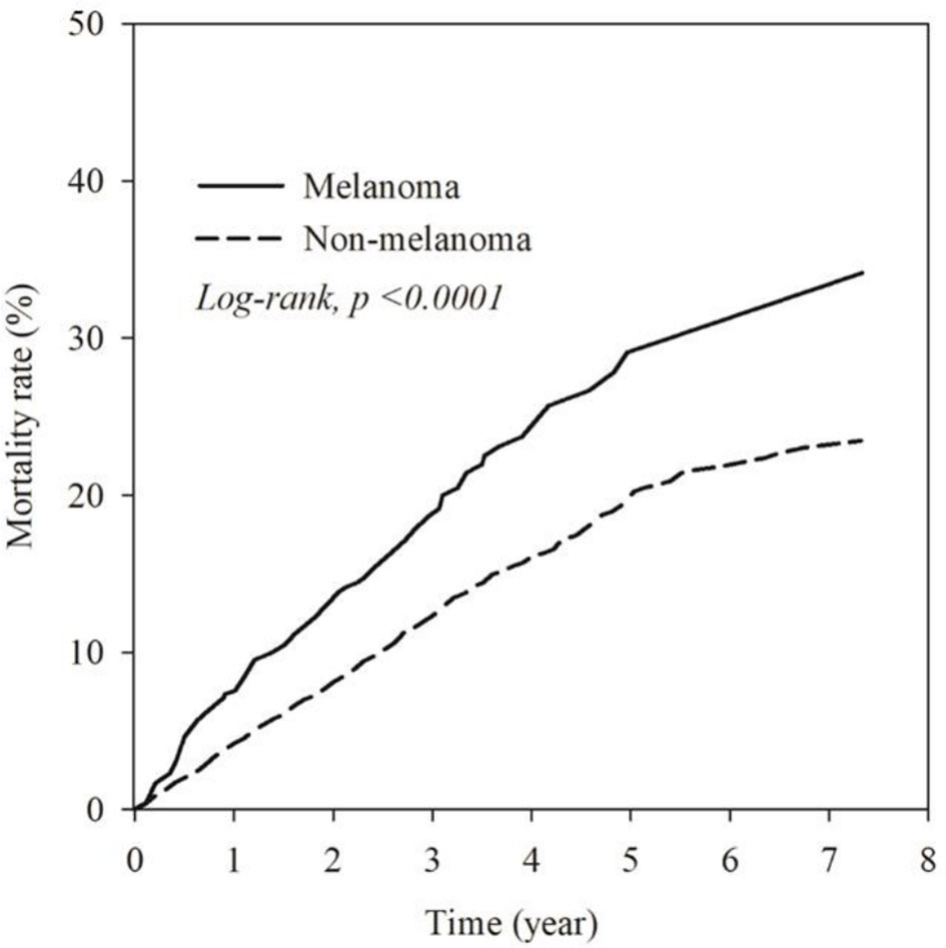
Kaplan-Meier plot for the mortality rate in melanoma group and non-melanoma group.

**Figure 3:**
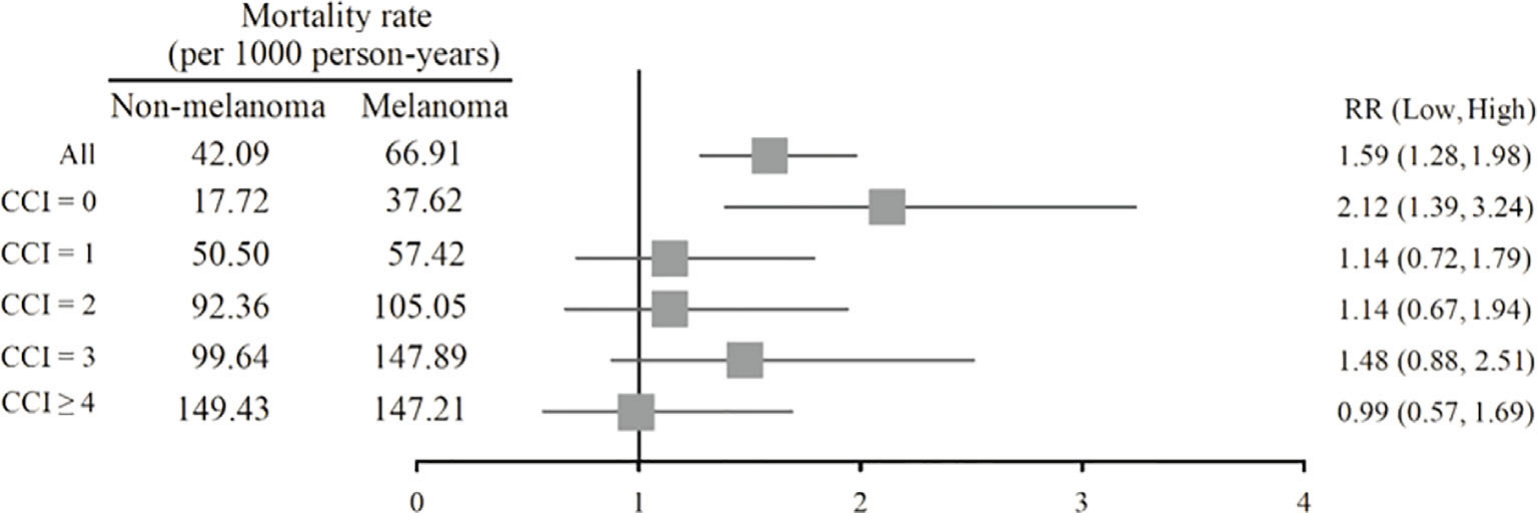
Forest plot of mortality rate according to CCI subgroups.

The mortality rate of the melanoma group in the Kaplan–Meier plot was significantly higher than that in the non-melanoma group (**Figure 2**). The mortality rate increased with increasing comorbidity in both cohorts and was higher among melanoma patients (**Figure 3**). The mortality rate for melanoma patients with comorbid score 1 was 57.42 per 1,000 person-years after diagnosis compared with 50.50 in the comparison cohort. In the most severe comorbidity group (≥4), the mortality rate was 147.21 per 1,000 person-years in the melanoma cohort compared with 149.43 in the comparison cohort. The mortality rate ratio (MRR) was 1.59 (95% CI: 1.28–1.98). The number of melanoma patients at stages 0, I, II, III, and IV was 52 (10.9%), 292 (61.3%), 106 (22.3%), 8 (1.7%), and 18 (3.8%), respectively (**Table 1**). In the univariate analysis, old age was associated with a significantly increased risk of death (p < 0.0001). Patients greater than and equal to 70 years of age (HR = 18.64, 95% CI: 10.96–31.72) had a worse prognosis than patients less than and equal to 54 years of age. The risk of death for people with comorbidity scores 1, 2, 3, and ≥4 was 2.65, 4.88, 5.48, and 7.77, respectively (**Table 2**). **Table 3** demonstrated the subgroup analysis of the risk of death between melanoma and non-melanoma groups. The risk of death for patients aged ≤54 between melanoma and non-melanoma group was the highest (HR = 13.08, 95% CI = 4.41–38.77, p < 0.0001) among the three age groups. For patients between melanoma and non-melanoma groups with comorbidity score 0, the HR risk of death was 2.40 (95% CI: 1.56–3.68, p < 0.0001). **Table 4** demonstrated the higher risk of mortality for people with comorbidity score 3 (HR = 2.79, 95% CI: 1.46–5.30, p = 0.002) and score ≥4 (HR = 2.75, 95% CI: 1.39–5.44, p = 0.004). Increased comorbidity was related to the advanced cancer stage at diagnosis. The HR risk of mortality in melanoma patients with comorbidity scores 1, ≥2 at stages 0 and I was 2.61 (95% CI: 1.01–6.78) and 2.62 (95% CI: 1.12–6.13), respectively. For patients with comorbidity scores 1, ≥2 at stage II, the HR of risk of mortality was 3.26 (95% CI: 1.20–8.84). The E-values for patients with comorbidity scores 1, ≥2 at stages 0 and I and comorbidity score ≥2 at stage II were 4.66, 4.68, and 5.97, respectively. We found in this study that the advanced stage of melanoma patients with more comorbidities was significantly associated with the higher risk of mortality rate.

**Table 2:** Cox proportional hazards model analysis for risk of death.

	Univariate		Multivariate^†^	
	HR (95% CI)	p value	HR (95% CI)	p value
Group				
Non-melanoma	Reference		Reference	
Melanoma	1.58 (1.27–1.97)	<0.0001	1.46 (1.17–1.82)	<0.001
Age				
≤54	Reference		Reference	
55–69	2.67 (1.49–4.77)	0.001	2.21 (1.23–3.96)	0.008
≥70	18.64 (10.96–31.72)	<0.0001	11.76 (6.84–20.24)	<0.0001
Sex				
Female	Reference		Reference	
Male	1.15 (0.99–1.33)	0.078	1.10 (0.94–1.28)	0.246
Monthly income (NT$)				
<20,000	Reference		Reference	
20,001–40,000	0.90 (0.77–1.05)	0.186	1.09 (0.86–1.39)	0.473
>40,000	0.58 (0.46–0.74)	<0.0001	1.11 (0.88–1.42)	0.380
Residential region				
Northern	Reference		Reference	
Central	1.26 (1.04–1.51)	0.016	1.07 (0.88–1.30)	0.503
Southern	1.19 (0.99–1.43)	0.061	1.07 (0.89–1.29)	0.487
Others	0.90 (0.67–1.20)	0.471	0.81 (0.60–1.09)	0.158
Hypertension	2.22 (1.91–2.58)	<0.0001	1.09 (0.93–1.28)	0.304
Hyperlipidemia	0.66 (0.54–0.82)	<0.001	0.43 (0.35–0.54)	<0.0001
Charlson comorbidity index score				
0	Reference		Reference	
1	2.65 (2.15–3.26)	<0.0001	1.99 (1.61–2.47)	<0.0001
2	4.88 (3.92–6.09)	<0.0001	3.13 (2.49–3.93)	<0.0001
3	5.48 (4.26–7.04)	<0.0001	3.36 (2.59–4.35)	<0.0001
≥4	7.77 (6.19–9.75)	<0.0001	4.77 (3.75–6.06)	<0.0001

^†^Adjusted for age, sex, hypertension, hyperlipidemia, monthly income, residential region, and Charlson comorbidity index score.

**Table 3:** Subgroup analysis of risk of death between melanoma and non-melanoma group.

	Non-melanoma		Melanoma		HR^†^ (95% CI)	p value
	N	No. of death	N	No. of death		
Age						
≤54	710	7	71	7	13.08 (4.41–38.77)	<0.0001
55–69	1,430	47	143	14	2.85 (1.55–5.23)	<0.001
≥70	2,620	571	262	72	1.33 (1.04–1.70)	0.024
					p for interaction	<0.0001
Sex						
Female	2,060	235	206	39	1.63 (1.16–2.29)	0.005
Male	2,700	390	270	54	1.50 (1.13–2.00)	0.006
					p for interaction =	0.6331
Monthly income (NT$)						
<20,000	1,527	231	144	34	1.76 (1.22–2.54)	0.003
20,001–40,000	2,349	320	235	44	1.33 (0.97–1.83)	0.078
>40,000	884	74	97	15	1.93 (1.09–3.42)	0.024
					p for interaction =	0.3457
Residential region						
Northern	1,989	236	242	42	1.55 (1.12–2.16)	0.009
Central	1,116	172	87	17	1.29 (0.78–2.13)	0.322
Southern	1,212	171	111	27	1.82 (1.20–2.74)	0.005
Others	443	46	36	7	1.37 (0.61–3.09)	0.447
					p for interaction =	0.775
Charlson comorbidity index score						
0	2,663	158	212	25	2.40 (1.56–3.68)	<0.0001
1	1,024	156	126	21	1.22 (0.77–1.93)	0.394
2	478	124	54	15	1.01 (0.58–1.76)	0.977
3	280	75	45	17	1.67 (0.98–2.86)	0.060
≥4	315	112	39	15	1.20 (0.70–2.08)	0.509
					p for interaction =	0.0799
Charlson comorbidity index score						
0	2,663	158	212	25	2.40 (1.56–3.68)	<0.0001
1	1,024	156	126	21	1.22 (0.77–1.93)	0.394
≥2	1,073	311	138	47	1.25 (0.92–1.71)	0.152
					p for interaction =	0.029

^†^Adjusted for age, sex, monthly income, residential region, hypertension, and hyperlipidemia.

**Table 4:** Cox proportional hazards model analysis for risk of mortality in melanoma patients.

	N	Numbers of death	HR^†^ (95% CI)	p value
Charlson comorbidity index score				
0	212	25	Reference	
1	126	21	1.24 (0.69–2.23)	0.475
2	54	15	1.58 (0.81–3.11)	0.184
3	45	17	2.79 (1.46–5.30)	0.002
≥4	39	15	2.75 (1.39–5.44)	0.004
**Stage 0, I**				
Charlson comorbidity index score				
0	155	8	Reference	
1	88	11	2.61 (1.01–6.78)	0.048
≥2	101	23	2.62 (1.12–6.13)	0.026
**Stage II**				
Charlson comorbidity index score				
0	45	12	Reference	
1	NA	NA	0.44 (0.14–1.33)	0.144
≥2	NA	NA	3.26 (1.20–8.84)	0.020
**Stage III, IV**				
Charlson comorbidity index score				
0	12	5	Reference	
1	NA	NA	3.86 (0.59–25.22)	0.158
≥2	NA	NA	5.17 (0.69–38.92)	0.110

^†^Adjusted for age, sex, monthly income, residential region, hypertension, and hyperlipidemia.

NA, not available.

## Discussion

In this nationwide registry-based cohort study, we included 476 men and women diagnosed with melanoma. According to tumor stage, 52 (10.9%) *in situ*, 398 (83.6%) had a localized tumor, 8 (1.7%) had regional metastases, 18 (3.8%) had distant metastases, and 364 (55.5%) suffered from one or more comorbidities (**Table 1**). Our study unraveled an association between a higher prevalence of comorbidities and a higher risk of death. The more comorbidity, the higher the risk of mortality. Comorbidity commonly is associated with poorer survival. Old age was associated with a significantly increased risk of death. In our study, patients greater than and equal to 70 years of age (HR = 18.64, 95% CI: 10.96–31.72) had a worse risk of death than patients 55–69 years of age (**Table 2**). There was an association between older age and more advanced tumors at diagnosis, leading to higher mortality among elderly people (34, 35). In Taiwan, malignant melanoma is an uncommon but fatal disease. One possible reason for low survival was that farmers delayed the diagnosis to old age. The comorbidities, or neglecting consciousness of melanoma, adverse effects of their treatment, or disguising the symptoms of the disease by the patient or doctor could be another possibility for late detection in people with comorbid conditions (36).

Gonzalez et al. (24) demonstrated that having comorbidity and cancer (colorectal, melanoma, breast, prostate) has resulted in late diagnosis of cancer. A previous study from Grann et al. (20) showed that comorbidity initiated a higher risk of complications and worse functional status, decreased quality of life, and poorer survival—especially in older patients. Houterman et al. (37) reported that increased levels of severe comorbidity led to less aggressive treatment that negatively influenced the survival of elderly patients aged 60–79 years. Comorbidities could worsen comorbid diseases and lower the functional status of metastatic melanoma patients receiving curative treatment (20). Research from Taiwan reported that a worse prognosis with great differences was mostly found in histologic subtypes, advanced stages, and acral lentiginous melanoma. Melanoma patients had a poorer prognosis when they were diagnosed with more advanced stage (26). Some studies suggested that coexistent disease was associated with worse survival and increased the possibility of being diagnosed with distant metastasis (8, 23, 38–43). Our study revealed the association between comorbidity and stage that may influence the prognosis of melanoma patients in Taiwan. **Table 4** showed that for people with comorbidity scores 3 and ≥4, HR of the risk of mortality was relatively higher than scores 1 and 2. The risk of mortality for patients with comorbidity score ≥2 at stages 0, I was a little higher than those with comorbidity score 1. The risk of mortality for patients with comorbidity score ≥2 at stage II was significantly higher than those with comorbidity score 1. Though the risk of mortality for patients with comorbidity score ≥2 at stages III, IV was lower than those with comorbidity score 1, no major differences were found because of too small a patient population.

Comorbidity might reduce survival because curative treatment is used less frequently in older patients. Consequently, survival of patients older than 70 years was not significantly influenced by comorbidity (44). Bradley et al. (45) reported that men with more comorbid conditions were less likely to receive treatment than those without comorbidities. The decision to receive treatment was determined mainly by the patient’s age, disease stage, tumor characteristics, and experience of the urologist (44, 46). Fowler et al. (47) revealed that associations between age and comorbidity were highly significant (p < 0.0001), as the age-adjusted risk of comorbid death was 5.7 times greater in men with severe compared to those without comorbidities. Two previous studies showed that less aggressive treatment for melanoma among patients with comorbidity may affect the mortality rate because of some of the interactions between melanoma and comorbidity. Decreased function of the immune system in older patients with more comorbidities may result in higher mortality because of the interaction between comorbidity and melanoma (48, 49). Koppie et al. (50) also reported that the conditions required for treatment, such as a history of comorbidities, age, performance status, and other related factors, are important for melanoma patients when they plan to receive necessary treatment. Generally, elderly patients with more comorbidities are less likely to receive the most aggressive chemotherapy combinations for avoiding a high risk of significant morbidity (50).

The minority of melanoma patients (44.5%) had none of the selected comorbidities since their melanoma was diagnosed. In the remaining 55.5% of the melanoma cohort with some prevalent comorbidity, the most common comorbid diseases were diabetes without chronic complication, chronic pulmonary disease, peptic ulcer disease, and cerebrovascular (**Table 1**). Among melanoma and non-melanoma groups, the proportion diagnosed with severe comorbidity increased with an increasing mortality rate, and the level of comorbidity was strongly associated with mortality (**Figure 3**).

However, the weight of the association differs by specific comorbid disease, patient age, cancer characteristics, and overall comorbidity burden (23, 34, 35). Comorbidity can be measured by counting the number of coexisting illnesses diagnosed in a cancer patient or by using a comorbidity index that integrates the number and severity of the comorbidity (51). Nowadays, the CCI is the most commonly applied index for comorbidities in cancer patients. The CCI score is the sum of weights of a patient’s coexisting conditions based on 19 disease categories. The weights originated from relative risk assessment acquired from a regression model. They are usually assigned from 1 to 6 points and then collapsed into categories of 0 point, 1 to 2 points, 3 to 4 points, and 5 or more points, respectively. The CCI has been previously verified as a prognostic method of comorbidity for some index cancers (31).

A study previously showed that both the survival and treatment of patients were affected by age and the extent of comorbidity. Racial differences in survival were greatest for patients without comorbidities and less pronounced at higher levels of comorbidity. Comorbidity elicited differential impact for prognosis, treatment, and survival (52). However, the outcomes of this study by using the CCI score were similar with many reports in Western countries (24, 39). Comorbidities that influence prognosis seriously may differ between Eastern and Western countries. We expect that future research can focus on which of these comorbidities may most seriously affect the prognosis of melanoma patients.

There is a limitation in this study. From Taiwan’s NHIRD, we selected random samples of 2 million from 23 million beneficiaries. Because melanoma is an unusual illness in Taiwan, the sample size of melanoma patients was relatively small in our research. However, NHIRD is a randomly selected representative sample of Taiwan’s general population. This may avoid sampling deviation or selection bias and provide more nationwide information in this study.

In conclusion, melanoma patients in Taiwan with comorbidity were associated with poorer survival. The level of comorbidity was robustly associated with the mortality rate. The presence of severe comorbidity was associated with an advanced stage of melanoma. The mortality rate was higher among patients with more comorbidities, and the influence of comorbidity varied by stage. Old age was associated with a significantly increased risk of death. A higher risk of mortality was found in patients who had localized tumors, regional metastases, or distant metastases with more comorbidity scores. Our study demonstrated that comorbidity and stage had an impact on the prognosis of Taiwan melanoma patients.

## Data Availability Statement

The data in this study we used were from the National Health Insurance Research Database and Taiwan Cancer Registry.

## Author Contributions

All authors listed have contributed to the work and approved its publication. Contributors: All other authors (YSH, PNC, SCC, JYH, YHW and JCCW) provided their input by contributing to the conceptualization. CKC contributed to the editing of the article.

## Conflict of Interest

The authors declare that the research was conducted in the absence of any commercial or financial relationships that could be construed as a potential conflict of interest.

## Publisher’s Note

All claims expressed in this article are solely those of the authors and do not necessarily represent those of their affiliated organizations, or those of the publisher, the editors and the reviewers. Any product that may be evaluated in this article, or claim that may be made by its manufacturer, is not guaranteed or endorsed by the publisher.
