# Supplementary material for: A Cohort Study: Comorbidity and Stage Affected the Prognosis of Melanoma Patients in Taiwan
Source: Front Oncol. 2022 Mar 3;12:846760. doi: 10.3389/fonc.2022.846760 (PMC8927660; doi:10.3389/fonc.2022.846760)
Supplement: Supplementary file 1 [file DataSheet_1.pdf]

Supplementary table 1

Comorbidity	Weight	ICD-9-CM	ICD-10-CM
Myocardial infarction	1	410.x<br>412.x	I21, I22, I252
Congestive heart failure	1	428.x	I50
Peripheral vascular disease	1	441.x<br>443.9<br>785.4<br>V43.4	I71, I790, I739, R02, Z958, Z959
Cerebrovascular disease	1	430.x-438.x	I60, I61, I62, I63, I65, I66,G450, G451, G452, G458, G459, G46, I64, G454, I670, I671, I672, I674, I675, I676, I677 I678, I679,I681, I682, I688, I69
Dementia	1	290.x	F00, F01, F02, F051
Chronic pulmonary disease	1	490.x-496.x<br>500.x-505.x	J40, J41, J42, J43, J44, J45, J46, J47, J67, J44, J60, J61, J62, J63, J66, J64, J65
Connective tissue disease	1	710.0<br>710.1<br>710.4<br>714.0-714.2<br>714.81<br>725.x	M32, M34, M332, M053, M058, M059, M060, M063, M069, M050, M052, M051, M353
Peptic ulcer disease	1	531.x-534.x	K25, K26, K27, K28
Mild liver disease	1	571.2<br>571.4-571.6	K702, K703, K73, K717, K740, K742, K746, K743, K744, K745
Diabetes without chronic complication	1	250.0-250.3<br>250.7	E109, E119, E139, E149, E101, E111, E131, E141, E105, E115, E135, E145
Diabetes with chronic complication	2	250.4-250.6	E102, E112, E132, E142 E103, E113, E133, E143 E104, E114, E134, E144
Hemiplegia or paraplegia	2	342.x<br>344.1	G81 G041, G820, G821, G822
Renal disease	2	582.x<br>583-583.7<br>585.x<br>586.x<br>588.x	N03, N052, N053, N054, N055, N056, N072, N073, N074, N01, N18, N19, N25

Any malignancy, including lymphoma and leukemia, except malignant neoplasm of skin	3	140.x-172.x<br>174.x-195.8<br>200.x-208.x	C0, C1, C2, C3, C40, C41, C43, C45, C46, C47, C48, C49, C5, C6, C70, C71, C72, C73, C74, C75, C76, C80, C81, C82, C83, C84, C85, C883, C887, C889, C900, C901, C91, C92, C93, C940, C941, C942, C943, C9451, C947, C95, C96
Severe liver disease	3	572.2-572.8	K729, K766, K767, K721
Metastatic solid tumor	6	196.x-199.1	C77, C78, C79, C80
AIDS/HIV	6	042.x-044.x	B20, B21, B22, B23, B24
